# Comparison of Different Solid-Phase Microextraction Formats Dedicated to the Analysis of Volatile Compounds—A Comprehensive Study

**DOI:** 10.3390/molecules29215137

**Published:** 2024-10-30

**Authors:** Martyna Natalia Wieczorek

**Affiliations:** Faculty of Food Science and Nutrition, Poznań University of Life Sciences, Wojska Polskiego 31, 60-624 Poznań, Poland; martyna.wieczorek@up.poznan.pl

**Keywords:** TF-SPME, odorants, extraction

## Abstract

The coupling of Solid-Phase Microextraction (SPME) technology with gas chromatography (GC) has a well-established and successful history. Traditionally, SPME fibers have been the most popular form thanks to their versatility and the ease with which they can be fully automated. However, alternative geometries for SPME have been developed over the years, beginning with Stir Bar Sorptive Extraction (SBSE) and later evolving into Thin-Film SPME (TF-SPME) devices. Each of these formats offers distinct advantages and disadvantages, which are explored in this study. The primary objective of this study was to comprehensively compare available forms of SPME devices, with a special focus on the advantages of TF-SPME, a novel microextraction method particularly suited for the analysis of odorants in food. The study involved analyzing a standard mixture of 11 key food odorants, representing a range of polarities, to evaluate the efficiency of TF-SPME devices in terms of the number of analytes extracted. Furthermore, four types of TF-SPME devices were compared against each other in both standard mixtures and actual food samples. The final stage of the study employed GCxGC-ToFMS analysis to showcase the potential of the most efficient HLB-TF-SPME device in the non-targeted analysis of complex samples, exemplified by unfiltered wheat beer. This analysis demonstrated the significant capability of HLB-TF-SPME in capturing and identifying a wide range of volatile compounds in complex matrices.

## 1. Introduction

Solid-phase microextraction (SPME) is a sample preparation technique used primarily in analytical chemistry for the isolation and concentration of analytes from various matrices, including liquid, solid, and gaseous samples. It was developed in the early 1990s by Pawliszyn and co-workers [1]. The core principle of SPME is the partitioning of analytes between the stationary phase and a sample matrix. The analytes in the sample are concentrated on the SPME fiber, which is later introduced into the analytical instrument for detection. Therefore, the whole extract present on the surface of the SPME device is simultaneously desorbed and subjected to analysis. Multiple advantages of this technique have been observed over the years, such as its solvent-free sample preparation, simplicity, sensitivity, versatility, and the possibility of on-site analysis. The innovative design of SPME features a fused silica fiber coated with a thin stationary phase layer to absorb or adsorb the analytes from the sample. The major SPME variant used in analytical chemistry was initially designed for combination with gas chromatography methods due to the possibility of thermo-desorption. Over the years, SPME stopped being exclusively tied to GC, as it was also coupled with liquid chromatography (LC) via liquid desorption before the extraction.

Additionally, alternatives to fiber geometries have been developed over the last 30 years. These included thin films and stir bar sorptive extraction (SBSE). These geometries were aimed at enlarging the sorption area of SPME devices, enhancing the method’s sensitivity. Many attempts to use TF-SPME devices in combination with gas chromatography have been made since SPME’s invention, such as spreading the material on glass [2], aluminum foil [3], etc. However, these approaches were not stable for a longer period and, therefore, not suitable for more than “in-house application”. The game changer in the area of TF-SPME was the application of carbon mesh as the support for the sorbent. Four types of TF-SPME are commercially available: HLB/PDMS, DVB/PDMS, CAR/PDMS, and pure PDMS. The advantage of TF-SPME devices over SBSE is the presence of the solid sorbent, which enhances the extraction of a wide range of analytes.

This study focuses on the demonstration of the TF-SPME potential in comparison with alternative SPME formats, a comparison of TF-SPME devices with different sorbents, and finally, the performance of these devices in non-targeted studies on beer samples.

## 2. Results

### 2.1. Comparison of Different SPME Forms: TF-SPME, SPME Fibers, and Stir Bars

Existing forms of Solid-Phase Microextraction (SPME) can be distinguished by their fiber coatings, geometry, and specific applications. Commercially available SPME fibers use a variety of coating materials, such as polydimethylsiloxane (PDMS), divinylbenzene (DVB), and carboxen (CAR). These materials are widely used for their versatility in different analytical contexts. Alternative materials for SPME purposes were also demonstrated for their efficiency, such as molecularly imprinted polymers, ionic liquids, covalent organic frameworks, etc. [4]. However, the majority of these alternative coatings are dedicated to liquid chromatography applications due to their lack of resistance to high temperatures in the GC injection port; therefore, thermal desorption used in gas chromatography is not an option there. This paper did not analyze these coatings, merely focusing on the SPME techniques dedicated to GC-related analysis.

Coatings applied to the SPME fibers are used in thin-film SPME (TF-SPME) devices. However, TF-SPME offers a significant advantage over traditional fibers thanks to the use of hydrophilic-lipophilic balance (HLB) particles as an alternative coating material. The use of HLB particles in the thin-film format enhances the sensitivity of the device toward a wide range of analytes [5]. This improvement is particularly beneficial for the extraction of polar analytes. Polar moieties in the sorbent structure improve efficiency without requiring additional techniques, such as derivatization or salting-out strategies, which are often necessary with traditional SPME fibers. However, the primary advantage of TF-SPME over fiber-based methods is the larger volume of the sorbent, which is distributed as a thin layer on the surface of a carbon mesh. This characteristic contributes to the superior performance of TF-SPME devices, aligning with the theoretical principles of extraction techniques. As shown in Equation (1) below, the amount of analyte extracted over time (*d_n_*/*d_t_*) is directly proportional to the surface area (*A*), assuming all other factors remain constant [1].

(1) $$\frac{d_{n}}{d_{t}}=C_{s}\left(\frac{D_{S}A}{\delta }\right)$$

The larger sorption area and advanced coating options available in the case of TF-SPME, particularly the use of HLB particles, facilitate enhanced sensitivity and more efficient extraction of polar analytes compared to traditional SPME fibers. This makes TF-SPME a powerful tool in analytical applications, where sensitivity and efficiency are critical.

On the other hand, SBSE uses a thick PDMS layer as a coating, which is known for its affinity towards non-polar compounds and relatively long equilibration times due to the thick layer of the coating. Another important limitation of SBSE is its low recovery of highly polar solutes (log Kow < 2) [6]. While the trend in analytical chemistry is to develop “universal” methods that can extract the widest possible range of compounds, the above-mentioned extraction properties of stir-bars are a limitation factor of this method. Some strategies were applied to enhance the applicability of this method to include more polar substances, such as adding sodium chloride to the sample. However, these strategies were not discussed in this study since only unmodified samples were analyzed.

**Figure 1:**
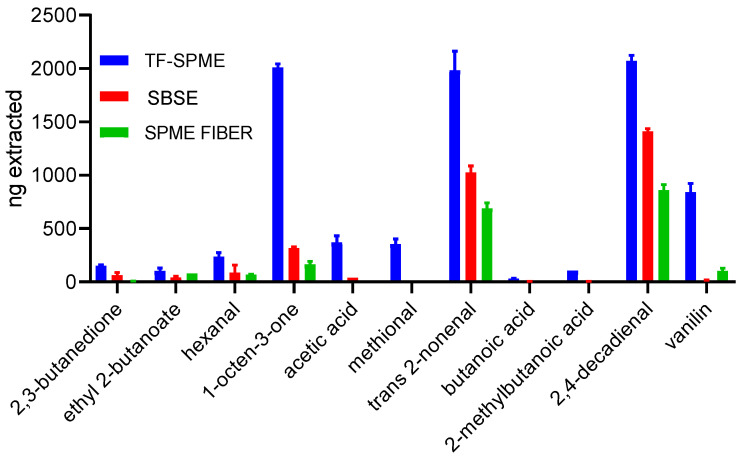
The comparison of extraction efficiency for three different SPME formats. Efficiency is expressed as the ng extracted.

In the first part of the study, three SPME techniques were compared: traditional SPME fibers, SBSE, and the latest TF-SPME (with HLB/PDMS coating) devices. As shown in Figure 1, the TF-SPME devices consistently outperformed the other two methods across all analytes tested. The experiments were conducted using a concentration of 200 ppb, with extraction carried out in direct mode, meaning that the devices were placed directly in water. They were compared based on the analysis of compounds selected after the literature review [7] as the odorants most commonly found in food products. These substances are the target compounds in most aroma-related studies; therefore, they were chosen as the targeted analytes. Extraction was performed in the direct mode by placing the TF-SPME devices directly in the liquid sample since this mode was reported to be more efficient for the extraction of a wide range of analytes [8]. In every case, TF-SPME demonstrated a clear advantage in extraction efficiency.

In the field of thin films, as with fibers or stir bars, extraction efficiency depends significantly on the extraction mode used in the study. It was demonstrated earlier that the direct mode (DI) demonstrated better overall performance [8]; therefore, it was the method of choice in this study. In most food-related research, headspace mode is the preferred choice. This preference is likely thanks to its convenience and simplicity, as well as its ability to reduce the risk of fiber contamination from the complex food matrix. However, as mentioned in the previous paragraph, in this study, the direct mode was chosen to present the best performance abilities of the investigated techniques. As shown in Figure 1, TF-SPME devices demonstrated significantly better results than alternative extraction methods for all 11 analyzed compounds. The best efficiency of this method is observed for the non-polar compounds, such as 1-octen-3-one, 2-nonenal, or 2,4-decadienal; however, extraction performance for these analytes is relatively good in the other two methods since hydrophobic compounds are rather easy to extract. The only exception was ethyl 2-butanoate, which is characterized by a high log *p* value of: 2.2 (see Table 1), with the observed performance not being as efficient as for the other hydrophobic compounds. Therefore, what it is worth noting is the extraction efficiency of the polar substances, such as acetic and butanoic acids, or 2,3-butanedione, which is notably better than after the application of SPME fibers or SBSE extractions. It is also worth highlighting that TF-SPME was the only method that made it possible to detect methional in the mixture.

Research on the application of TF-SPME devices in flavor and fragrance studies is limited, though some articles have been published. Stuff and colleagues utilized carbon mesh TF-SPME as an extraction tool for odorants in beverages, but no comparisons with alternative methods were provided [9]. There have also been developments in combining TF-SPME with stir bars in a single extraction workflow, leveraging their complementary abilities to cover compounds with different polarities. Authors demonstrated that placing both devices in one desorption liner after simultaneous extraction from a single vial increased sensitivity and extended the linearity range [10].

Studies that compared TF-SPME with alternative methods mostly focused on early versions of TF-SPME, which were not stabilized on the durable carbon mesh material. Instead, these early formats were self-supported or used glass or aluminum foil as support. While they showed improved performance over fiber-based methods, their widespread application was hindered by a strong siloxane background and limited stability [11,12,13].

When comparing these three devices, it is important to consider the economic aspects. The use of SPME fibers in routine laboratory analysis does not require any costly equipment apart from a relatively inexpensive injection liner. In contrast, employing stir bars or TF-SPME devices necessitates a thermal desorption unit (TDU) to introduce the extract to the system, which can lead to significant initial expenses when incorporating these techniques into laboratory routines.

Although the unit prices of these devices are comparable, there are differences in their reusability. SPME fibers and stir bars are known to be suitable for multiple uses, while ongoing research suggests that TF-SPME devices may also be reusable, although the results have yet to be published. One major advantage of SPME fibers is the availability of fully automated extraction systems. However, despite their reusability, SPME fibers are relatively fragile compared to the more durable TF-SPME devices and stir bars.

Considering all these factors, an appropriate laboratory technique should be carefully selected based on the specific requirements and economic considerations of each laboratory.

### 2.2. Comparison of Commercially Available TF-SPME Devices

In the second part of this study, we compared all types of TF-SPME devices available commercially, i.e., pure PDMS, HLB/PDMS, CAR/PDMS, and DVB/PDMS.

**Figure 2:**
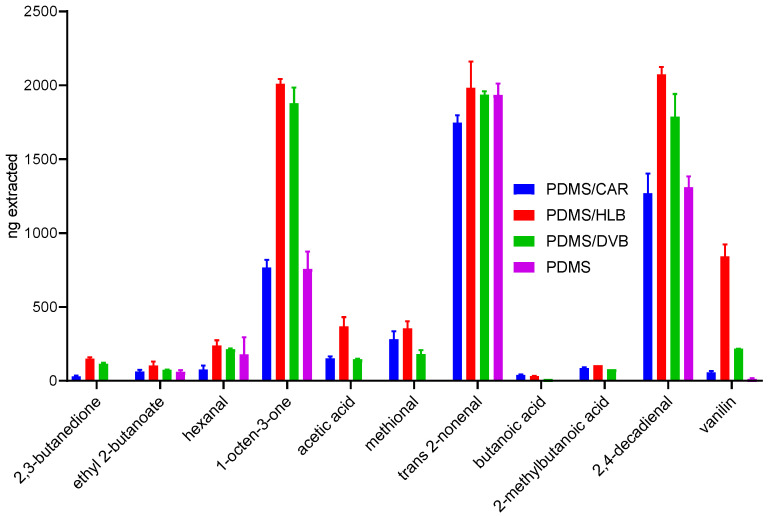
Comparison of different TF-SPME devices. Four commercially available TF-SPME devices were compared: PDMS/CAR, PDMS/HLB, PDMS/DVB, and PDMS.

The comparison between different TF-SPME phases demonstrated the best performance after extraction with the HLB-TF-SPME device in the case of all the analyzed compounds. The greatest benefits of using HLB TF-SPME devices were observed in the case of polar analytes, such as acetic acid, 2,3-butanedione, or vanillin (see Figure 2). The HLB particles contain lipophilic divinylbenzene, which is highly efficient in the extraction of non-polar analytes, and at the same time, the hydrophilic n-vinylpyrrolidone [14], which is the moiety for the extraction of polar compounds. Therefore, as mentioned above, the efficiency of polar compound extraction is notably better than that of TF-SPME with pure DVB sorbents. The HLB extraction material has not been popular in recent years for GC applications, mainly due to the lack of commercially available fibers containing this material. This material for SPME arrows was introduced by Yu et al. to extract microbial metabolites and fatty acids from salmon filets [15]. Worth noting is the high extraction efficiency for non-polar analytes by all types of TF-SPME devices. The results observed for most hydrophobic compounds, such as trans-2-nonenal (log *p* 3.1), obtained after extraction by all four types of TF-SPME devices were similar. At the concentration of analytes of 200 ppb, while the volume of the analyzed sample was 10 mL, the amount spiked in the sample was 2000 ng. Therefore, based on the results presented in Figure 2, we can conclude that the extraction performance was close to exhaustive for such compounds as 1-octen-3-one, trans-2-nonenal, or 2,4-decadienal. It might be an important factor influencing the linearity range in complex samples since it might reduce the competition phenomena [8]. For other compounds with a more hydrophilic character, performance was notably poorer. An important observation is also the fact that no carryover was observed in the analysis. As demonstrated in this paper, the larger area of TF-SPME compared to SPME fibers can itself improve the linearity range in quantitative analysis; however, if the obtained linearity range still fails to provide satisfactory results in complex matrices, it is advisable to use already developed approaches [8,16].

### 2.3. Comparison of TF-SPME Performance in Beer Samples

The next step to compare the TF-SPME devices comprised an analysis of beer samples subjected to the analysis by four types of TF-SPME in a direct mode. Beer was selected as the matrix to evaluate the performance of TF-SPME devices for several reasons. Firstly, there is a research history of applying TF-SPME to this matrix [8]. Secondly, brand-name beer is relatively consistent in composition, which is important for achieving good reproducibility in this type of study. Additionally, as a liquid sample, beer was well-suited for demonstrating the performance of TF-SPME devices in liquid samples. However, it should be noted that this choice also represents a limitation of the study, as it does not assess the technique’s performance across a wider range of sample types. Therefore, further research on different matrices is planned to fully explore and develop the potential of this technique.

**Figure 3:**
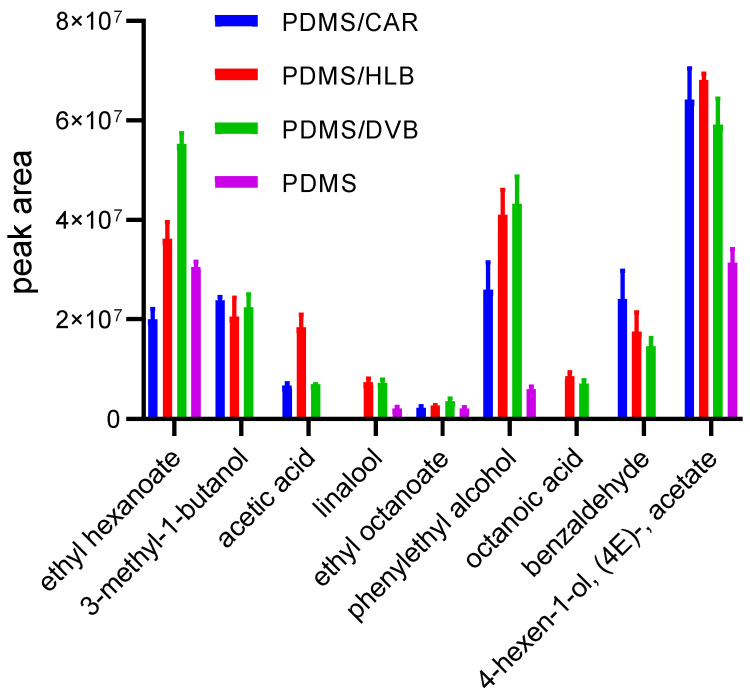
A comparison of extraction performance of volatiles from beer samples by four different TF-SPME devices.

A total of nine compounds identified in analyzed beer were analyzed and compared between TF-SPME devices (Figure 3). They were selected based on their polarity differences and abundance in beer samples. The analyzed compounds included ethyl hexanoate, 3-methyl-1-butanol, acetic acid, linalool, ethyl octanoate, phenethyl alcohol, octanoic acid, benzaldehyde, and 4-hexenyl acetate. These compounds differ in their properties, such as volatility and polarity. According to the data presented, the performance of compared TF-SPME in the real samples demonstrated the similar extraction efficiency of the DVB TF-SPME and HLB TF-SPME devices for the above-mentioned analytes. However, some differences were observed, such as the better efficiency of ethyl hexanoate extraction by DVB compared with HLB, which might be related to its non-polar character (log *p*: 2.4), as DVB particles exhibit better affinity toward non-polar compounds.

However, due to polar moieties in its structure, the HLB-TF-SPME demonstrated better results for acetic acid extraction (log *p*: −0.2). In turn, CAR TF-SPME demonstrated less effective results compared with DVB-TF-SPME and HLB-TF-SPME. The extraction was performed three times, and a good RSD was observed; nevertheless, it is planned to perform further studies on TF-SPME’s reusability.

### 2.4. Non-Targeted Analysis of Wheat Beer Volatiles by HLB TF-SPME GCxGC-ToFMS

In the final step of this study, an analysis was performed on a more complex sample, which was wheat beer from the Polish brand “Żywiec” (Heineken Group, Żywiec, Poland). The analysis used the HLB-TF-SPME device, as it previously demonstrated the most efficient results.

The TF-SPME GCxGC-ToFMS analysis made it possible to identify 139 volatiles in the wheat beer sample. They were divided into six groups, namely alcohols, aldehydes, esters, alkanes, alkenes, ketones, acids, and other compounds. In each group, the volatiles from a wide polarity spectrum and volatility were extracted by TF-SPME devices, with the log *p*-value of the extracted compounds starting at −1.76 (glycerin) and ending at 15.18 (heptacosane). Therefore, it was demonstrated that the method shows good potential as an option for the extraction of a wide spectrum of analytes.

The number of analytes identified in wheat beer after GCxGC-ToFMS analysis is large (Table 2). Many of these substances possess some aroma activity; therefore, potentially they might be responsible for the aroma of the analyzed beer. This paper focused on the analysis of key odorants in wheat beer; 16 compounds were identified as “most odor-active” in Bavarian Wheat Beer. In this study, a Polish brand of wheat beer was analyzed. Therefore, fewer odorants might be presented there, and key odorants also probably differ from those in German beer [17]. Still, many of them were present in the chromatogram, such as 3-methylbutanol, 3-methylbutyl acetate, ethyl hexanoate, ethyl octanoate, acetic acid, linalool, and many other important ones. Some of these key odorants identified in this paper were not detected by TF-SPME-GCxGC-ToFMS, such as 2-acetylpyrroline or methylpropanol, along with a few others. However, this may have been caused either by the fact that the analyzed beer was a different brand than the above-mentioned paper or simply by the still too-low sensitivity of the applied method. Nevertheless, if they were present, they could probably be detected using the targeted approach with a different detector in either the MRM or SIM mode.

One additional finding observed in the analysis was the low background related to the coating of the TF-SPME device. It was mentioned before by Grandy et al. that the novel idea of spreading TF-SPME slurry in the carbon mesh, as a result, generates less membrane bleed and is associated with its siloxane background. In this study, no membrane bleeding was observed in the GCxGC-ToFMS chromatogram. Therefore, it seems that the large sorbent volume does not significantly affect the chromatogram quality, which had been a considerable concern in the case of the TF-SPME method.

## 3. Materials and Methods

### 3.1. Samples and Reagents

All standards used in the experiments were obtained from Merck (Poznań, Poland). Individual standard solutions, as well as stock solutions, were prepared in ethanol. Beer samples were purchased from local shops, and each was opened, degassed using a Whatman paper filter, and then transferred to vials for freezing. Before analysis, the beer samples were thawed and brought to room temperature. Each analysis required 10 mL of beer, so a single bottle was sufficient for the one step of the experimental process.

### 3.2. Extraction Procedure

Extraction was conducted using 10 mL SPME vials, with the appropriate TF-SPME holder provided by Gerstel (Mulheim, Germany). The extraction process was run on a Gerstel Agitator, operating at 400 rpm and maintaining a controlled temperature of 40 °C. After each direct immersion extraction in beer, the TF-SPME device was placed in water and agitated for 60 s. This rinsing step was applied only to the beer samples to remove non-specific residues, such as sugars and peptides, from the TF-SPME surface; it was deemed unnecessary during experiments with standards.

After rinsing with water, the TF-SPME was placed in a 5 mL vial containing MilliQ water (Merck, Poznań, Poland) and vortexed for 30 s before the TF-SPME devices were dried with Kimwipes to ensure that water residues had been completely removed from the membrane. Later, the membranes were placed in desorption tubes dedicated to TDU units and analyzed via GC-MS. The nanograms extracted were calculated based on technical calibration curves. According to the procedure described earlier [13]. On-membrane liquid injection was performed on a PDMS thin film held in place with locking tweezers, while a gas-tight syringe was used to deliver 2 µL of standard solution on the uncoated edge of the membrane. Later, the TF-SPME device was placed in the desorption tube, and analysis was started. The membranes used in this study were prepared according to the procedure described earlier [18]. All analyses were performed in triplicates.

### 3.3. Analysis by GC-MS

The GC-MS instrument consisted of a 7890A/7000B, Agilent Technologies, Santa Clara, CA, USA) GC and an Agilent Technologies TripleQuadrupole MS (Santa Clara, CA, USA). The GC system was connected to a Gerstel MPS2 autosampler for membrane desorption and injection, together with a thermal desorption unit (TDU) and a GERSTEL-cooled injection system (CIS). The GC system was equipped with a DB-FFAP (30 m × 0.25 mm × 0.25 um) column. Helium was used as a carrier gas, and its flow was set at 0.8 mL/min; the oven temperature was as follows: 40 °C (1 min), followed by 6°/min to 200 °C, and finally 25 °C/min to 280 °C (3 min). The temperature of the injector used for injection by SPME fibers was 230 °C. 

High-volume desorption from the TF-SPME devices and twisters was performed using TDU. The membranes were placed in glass desorption tubes designed for use with these devices. Desorption was run using the following temperature program: initial temperature of 60 °C and held for 3 min.

### 3.4. Analysis by GCxGC-ToFMS

HLB TF-SPME devices extracted the volatiles in wheat beer samples, which TDU-GCxGC-ToFMS later analyzed. The parameters of the TDU instrument were the same as those described in Section 2.2. After desorption, the volatiles were transferred to the DB-5 column (25 m × 0.2 mm × 0.33 μm, Agilent Technologies, Santa Clara, CA, USA) and Supelcowax 10 (1.2 m × 0.1 mm × 0.1 μm, Supelco, Bellefonte, PA, USA) was used as the second column. The gas used in the system was helium, with the flow rate set up at 0.8 mL/min. The primary oven temperature was programmed as follows: 40 °C for 1 min before rising to 6 °C/min to 200 °C, and afterward reduced at 25 °C/min to 235 °C, where it was held for 5 min. In the secondary oven, the following programming was used: held for 1 min at 65 °C before rising at 6 °C/min to 225 °C, followed by 25 °C/min to 260 °C, where it was held for 5 min. The transfer line temperature was 260 °C. The modulation time was 4 s. The time-of-flight mass spectrometer operated at a mass range of 38–388 *m*/*z,* and the detector voltage was 1700 V at 150 spectra/s. The total analysis time was 34 min. Analyses were performed in triplicate. Data were collected using the LECO Chroma, TOF v.4.44 software (St. Joseph, MI, USA). The identification was accomplished using the National Institute of Standards and Technology (NIST) library (version 2.0) of mass spectra obtained from highly sensitive ToFMS. For the analyte match criteria, the following parameters were used: minimum similarity = 70%, mass threshold = 10, and signal-to-noise ratio = 1000. Compounds detected in the extract were identified tentatively. Additionally, retention indexes were calculated and compared with respective data from the literature.

## 4. Conclusions

This study aimed to compare the most popular types of SPME devices in terms of their efficiency in extracting important food odorants. The odorants analyzed here differed in their physio-chemical properties, such as polarity and volatility. The study demonstrated that the novel form of SPME—TF-SPME devices performed significantly better when compared with the regular fibers or stir bars. In the next part of the experiment, performance was compared between different forms of TF-SPME, such as HLB/PDMS, CAR/PDMS, DVB/PDMS, and pure PDMS. These devices were investigated in terms of extraction from the standard mixture and a food sample such as filtered beer. The results demonstrated the best performance for the HLB/PDMS TF-SPME device. Therefore, in the final step of the experiment, the most efficient TF-SPME device was used to extract volatiles from a relatively complex sample, such as unfiltered wheat beer, and it was further analyzed using the GCxGC-ToFMS instrument. Results from the GCxGC analysis proved the outstanding potential of the TF-SPME devices, which allowed the extraction and further analysis of 139 compounds with a wide range of physicochemical properties. Based on data from the literature, the analysis allowed the majority of key odorants to be detected, even those with low log *p* values. Therefore, it was concluded that the TF-SPME devices are a novel format of SPME, exhibiting considerable potential as an alternative method dedicated to the extraction of volatiles from samples in which sensitivity issues are observed.

## Figures and Tables

**Table 1:** The list of analyzed compounds together with their log *p* (based on https://pubchem.ncbi.nlm.nih.gov; accessed on 1 June 2024).

Compound Name	log *p* Value
2,3-butanedione	−1.34
ethyl 2-butanoate	2.2
hexanal	1.8
1-octen-3-one	2.4
acetic acid	−0.2
methional	0.3
trans 2-nonenal	3.1
butanoic acid	0.8
2-methylbutanoic acid	1.2
2,4-decadienal	3.2
vanillin	1.2

**Table 2:** Main volatile compounds identified using GCxGC-ToFMS in wheat beer samples.

Alcohols	Log *p*	RI	Mean		sd
			^10^6^		
(S)-(+)-1,2-Propanediol	−0.9	1032	33.28	±	3.04
1-Butanol, 3-methyl-	1.2	744	16.21	±	13.80
1-Decanol	4.6	1257	5.73	±	0.64
1-Decanol, 2-hexyl-	7	1503	192.66	±	52.23
1-Dodecanol	5.1	1565	21.07	±	0.35
1-Eicosanol	9.5	2275	128.21	±	57.95
1-Heptanol	2.7	943	7.75	±	0.23
1-Hexadecanol	7.3	2166	0.51	±	0.07
1-Hexanol, 2-ethyl-	3.1	1013	6.36	±	2.93
1H-Indole-3-ethanol	1.5	1522	9.79	±	0.92
1-Nonanol	4.3	1154	11.70	±	3.75
1-Octanol	3	1077	6.93	±	4.96
1-Octanol, 2,7-dimethyl-	3.3	1350	0.60	±	0.03
1-Octen-3-ol	2.7	1066	3.45	±	0.64
1-Pentanol	1.4	804	37.12	±	43.95
1-Propanol, 3-(methylthio)-	0.8	1040	0.77	±	0.06
1-Tridecanol	5.1	1680	7.83	±	0.44
1-Undecanol	4.5	1530	5.04	±	0.62
1,6-Octadien-3-ol, 3,7-dimethyl-	2.7	1082	27.59	±	14.90
2-Ethyl-1-dodecanol	5.2	1620	26.55	±	4.60
2-Furanmethanol	0.3	1600	19.59	±	6.13
2-Methoxy-4-vinylphenol	2	1604	52.72	±	4.95
2-Methyl-1-undecanol	4.8	1611	0.78	±	0.07
2-Nonen-1-ol, (E)-	3.5	1222	0.35	±	0.01
2-Pentanol	1.1	720	1.07	±	0.01
2,3-Butanediol	−0.9	1200	56.94	±	2.04
2,6-Octadien-1-ol, 3,7-dimethyl-, (E)-	3.5	1227	7.50	±	0.38
3-Pentanol	1.2	735	3.52	±	0.85
4-Penten-2-ol	1.2	820	0.72	±	0.05
Isopropyl Alcohol	0.05	453	4.06	±	0.49
Phenol	1.5	978	4.11	±	1.46
Phenol, 2-butyl-	3.3	1301	1.07	±	0.11
Phenol, 3-ethyl-	2.6	1160	7.03	±	1.68
Phenylethyl alcohol	1.4	1080	43.84	±	38.51
Aldehydes					
2-Nonenal, (E)-	3.52	1160	1.59	±	0.05
2,4-Decadienal	4.13	1404	19.63	±	1.84
Cinnamaldehyde, (E)-	2.29	1380	18.61	±	0.57
Decanal	3.74	1202	3.83	±	0.34
Furfural	0.83	950	25.83	±	0.12
Heptanal	2.64	901	8.18	±	0.26
Nonanal	3.35	1120	9.17	±	0.28
Octanal	3.06	1001	29.50	±	5.50
Undecanal	4.06	1302	1.74	±	0.18
Esters					
1-Butanol, 3-methyl-, acetate	1.45	901	266.62	±	285.26
2-Butoxyethyl acetate	1.26	1200	0.60	±	0.03
2-Propenoic acid, 3-(4-methoxyphenyl)-, 2-ethylhexyl ester	5.6	2102	2.25	±	0.17
2-Propenoic acid, 3-phenyl-, ethyl ester	3.25	1521	1.19	±	0.09
á-Phenylethyl butyrate	3.5	1311	1.46	±	1.61
Acetic acid, 2-phenylethyl ester	2.52	1310	121.20	±	5.81
Acetic acid, hexyl ester	2.89	1150	1.67	±	0.08
Acetic acid, phenylmethyl ester	2.1	1251	6.72	±	0.32
Benzoic acid, 2-ethylhexyl ester	4.94	1466	6.06	±	1.07
Benzoic acid, 2-hydroxy-, butyl ester	3.70	1524	2.05	±	0.19
Benzoic acid, 2-hydroxy-, pentyl ester	4.1	1659	6.14	±	7.68
Benzoic acid, 2-hydroxy-, phenylmethyl ester	4.5	1773	9.10	±	0.56
Benzyl Benzoate	4.0	1911	6.04	±	0.29
Butanoic acid, 2-methyl-, ethyl ester	1.85	886	3.03	±	0.23
Butyric acid, 2,3-dioxo-, 2-methyloxime, ethyl ester	2.1	1117	5.61	±	0.42
Dodecanoic acid, 1-methylethyl ester	6.50	1781	1.82	±	0.09
Dodecanoic acid, ethyl ester	5.51	1643	0.42	±	0.02
Dodecanoic acid, methyl ester	4.86	1551	4.21	±	0.20
Formic acid, 2-phenylethyl ester	2.09	1253	5.50	±	0.26
Formic acid, octyl ester	2.75	1213	0.67	±	0.03
Hexanoic acid, ethyl ester	2.83	1012	11.14	±	0.53
Isopropyl myristate	7.34	2210	20.13	±	1.89
Methyl 2-furoate	1.13	1177	17.78	±	0.85
Methyl salicylate	2.55	1193	2.38	±	0.11
Octanoic acid, ethyl ester	3.69	1233	7.20	±	7.01
Oxalic acid, allyl hexadecyl ester	7.5	2309	7.89	±	0.59
Oxalic acid, allyl octadecyl ester	8.0	2502	7.26	±	5.44
Oxalic acid, allyl octadecyl ester	8.0	2411	7.10	±	0.53
Oxalic acid, isobutyl hexadecyl ester	7.8	2437	12.32	±	0.92
Oxirane, tetradecyl-	4.98	1522	30.35	±	38.52
Phthalic acid, butyl 4-octyl ester	7.5	2412	30.29	±	31.55
Propanoic acid, 2-hydroxy-, ethyl ester	−0.3	761	3.82	±	4.91
Alkanes					
Cyclohexane, (3-methylpentyl)-	5.56	1064	5.70	±	0.15
1-Cyclohexyl-1-(4-methylcyclohexyl)ethane	7.32	1722	0.14	±	0.01
Cyclooctane, 1,2-diethyl-	4.78	1173	13.34	±	0.58
Eicosane	11.4	2000	38.97	±	0.47
10-Heneicosene (c,t)	10.54	2314	6.18	±	4.65
Heptadecane	8.76	1711	42.87	±	43.21
Heptadecane, 2,6,10,14-tetramethyl-	9.37	1912	40.47	±	3.90
Nonadecane	9.96	1763	286.72	±	40.60
Octadecane	9.36	1803	7.74	±	0.28
Pentadecane	7.55	1505	30.80	±	0.52
Pentadecane, 2,6,10-trimethyl-	8.15	1589	29.23	±	9.22
Tetradecane	6.94	1411	9.04	±	0.55
Tridecane	6.33	1302	12.66	±	2.29
Tridecane, 2-methyl-	6.94	1333	0.47	±	0.03
Undecane	5.10	1104	3.49	±	1.03
Undecane, 3,8-dimethyl-	5.71	1159	7.01	±	0.55
Heptadecane, 2,6-dimethyl-	8.76	1744	11.23	±	1.71
Hexadecane	8.15	1605	110.19	±	10.26
Heptacosane	15.18	2711	21.20	±	24.62
Hexadecane, 4-methyl-	8.91	1649	4.99	±	0.03
Alkenes					
1-Docosene	11.04	2200	157.77	±	59.05
1-Dodecene	6.34	1201	1.03	±	0.09
1-Nonene	4.82	952	0.82	±	0.03
1-Octene, 3,7-dimethyl-	5.23	1000	1.93	±	0.41
1-Octene, 6-methyl-	5.21	993	0.70	±	0.06
1H-Phenalene	3.42	1643	2.80	±	0.20
5-Eicosene, (E)-	10.32	2241	0.50	±	0.01
Azulene	4.5	1700	2.25	±	0.21
Cyclohexene, 4-(4-ethylcyclohexyl)-1-pentyl-	6.18	1423	7.89	±	0.00
Pyrene	5.18	2511	0.19	±	0.01
Ketones					
2-Nonanone	3.63	1178	1.05	±	0.00
2-Undecanone	4.26	1294	11.97	±	1.03
Butyrolactone	−0.56	752	4.72	±	0.44
2H-Pyran-2-one, tetrahydro-6-methyl-	0.97	1205	8.57	±	1.28
3-Buten-2-one, 4-phenyl-, (E)-	2.6	1500	0.76	±	0.07
Other compounds					
1-Octadecyne	8.45	1911	27.93	±	1.99
1H-Benzotriazole, 4-methyl-	1.77	1349	3.12	±	0.19
Benzothiazole, 2-methyl-	2.52	1603	0.34	±	0.09
Dibenzofuran	3.76	1500	4.30	±	0.18
Dibutyl phthalate	4.5	2201	2.81	±	0.16
Furan, tetrahydro-	0.55	605	1.93	±	0.20
Glycerin	−1.76	611	17.94	±	0.52
Indole	2.14	1402	1.62	±	0.15
Levoglucosenone	−0.12	1233	7.27	±	0.13
Methoxyacetic acid, 2-propenyl ester	0.9	1100	1.98	±	0.61
Triacetin	−0.14	1004	13.31	±	0.93
Trimethylamine	0.36	400	11.13	±	2.30
Acids					
2-Propenoic acid, 3-phenyl-	2.15	1427	0.54	±	0.04
4-Ethylbenzoic acid	3.19	1734	6.56	±	0.04
Acetic acid	−0.17	840	9.79	±	13.36
Benzoic acid	1.87	1593	10.53	±	5.81
Benzoic acid, 3-methyl-	2.26	1630	0.49	±	0.05
Butanoic acid, 3-methyl-	1.92	947	4.69	±	0.35
Dodecanoic acid	2.41	1884	11.38	±	5.02
Formic acid	−0.54	640	19.78	±	0.35
Heptanoic acid	2.41	1389	86.59	±	0.95
Hexanoic acid	2.05	1279	73.86	±	66.00
n-Decanoic acid	4.09	1690	120.15	±	9.16
n-Hexadecanoic acid	7.23	2213	0.51	±	0.07
Nonanoic acid	3.46	1579	33.63	±	4.77
Octadecanoic acid	7.14	2295	20.00	±	6.75
Octanoic acid	2.92	1489	355.23	±	21.93
Oleic acid	7.64	2210	4.00	±	0.63
Tetradecanoic acid	5.9	2110	4.31	±	0.08

## Data Availability

Data will be available on request from author.
